# Comparison of Efficacy, Safety, and Economic Outcomes Between Biosimilar ABP 215 and Originator Bevacizumab in Japanese Patients With Colorectal Cancer

**DOI:** 10.7759/cureus.72260

**Published:** 2024-10-24

**Authors:** Takahiro Sumimoto, Ryota Tanaka, Ryosuke Tatsuta, Miki Kubota, Hiroki Itoh

**Affiliations:** 1 Department of Clinical Pharmacy, Oita University Hospital, Yufu, JPN

**Keywords:** bevacizumab, biosimilar, colorectal cancer, originator, therapeutical safety

## Abstract

Background

Bevacizumab is one of the most effective anticancer treatment options for patients with unresectable advanced or recurrent colorectal cancer. The high cost of the drug has been a barrier to its use, but in recent years, biosimilars with lower prices have been launched in Japan. This study compared the efficacy, safety, and cost-effectiveness of the originator and biosimilar products in Japanese patients with colorectal cancer.

Methods

This is a single-center, retrospective, observational cohort study including patients diagnosed with colorectal cancer who received the originator bevacizumab (Avastin®) or a biosimilar ABP 215 between January 2018 and April 2024. Enrolled colorectal cancer patients were divided into two cohorts: those who only received the originator and those who received only the biosimilar ABP 215 until the end of bevacizumab therapy. Efficacy was evaluated in terms of progression-free survival (PFS) over a two-year follow-up period. Adverse events were graded using the Common Terminology Criteria for Adverse Events (CTCAE) v5.0.

Results

A total of 159 patients were eligible for this study and divided into the originator cohort (n=121) and the biosimilar ABP 215 cohort (n=38). The Kaplan-Meier curves and a log-rank test showed no significant differences in median PFS between the originator and biosimilar ABP 215 cohorts (8.41 months [95% CI, 7.33-9.43] and 7.13 months [95% CI, 5.03-9.66], respectively, *p*= 0.460). The hazard ratio was 1.07 (95% CI, 0.89-1.29, p= 0.462). The incidence of any grade and grade ≥3 adverse events did not differ significantly between cohorts. Economic outcomes indicated a potential savings of approximately 800,000 Japanese yen per patient with biosimilar ABP 215 use.

Conclusions

Although this is a single-center, retrospective, observational study with limitations in terms of the number of cases and background factors, the use of the bevacizumab biosimilar ABP 215 product is recommended in Japan from the perspective of reducing medical costs, given the findings of no differences in efficacy and safety.

## Introduction

Colorectal cancer has the third-highest morbidity of all cancers worldwide and is the second-leading cause of cancer death in both men and women [1]. In Japan, the number of deaths owing to colorectal cancer has increased gradually, with an estimated 42.69 deaths per 100,000 people in 2021 [2]. It is the second-most common cancer after prostate cancer in men and breast cancer in women, and it has the highest annual morbidity rate for both combined [3].

The treatment strategy for colorectal cancer depends on the depth of primary tumor invasion, its stage, the feasibility of surgical resection, and mutation status. The optimal individual therapy is comprehensively determined by prolonging survival and suppressing tumor progression, improving quality of life, and alleviating tumor-related symptoms. Pharmacotherapies for colorectal cancer include adjuvant chemotherapy, to prevent recurrence after surgery, and anticancer therapies, for unresectable, advanced, or recurrent cancers, to prolong life and relieve symptoms. According to the 2019 Japanese Society for Cancer of the Colon and Rectum (JSCCR) guidelines for the treatment of colorectal cancer [4], regimens in combination with oxaliplatin or fluoropyrimidine monotherapy are recommended for the former. For the latter, immune checkpoint inhibitors alone, or fluoropyrimidine-based chemotherapy plus epidermal growth factor receptor or vascular endothelial growth factor (VEGF) antibodies, are selected based on the results of prior microsatellite instability testing and *RAS*/*BRAF* genetic testing.

Bevacizumab, the first anti-VEGF monoclonal antibody, was launched in 2004 under the brand name of Avastin®. Bevacizumab prevents VEGF from binding to its receptor, thereby suppressing angiogenesis, which is necessary for solid tumor cells to persist and grow [5]. The inhibition of VEGF also reduces vascular permeability and tumor interstitial pressure, allowing chemotherapeutic agents to be delivered more effectively to the target tumor [6,7]. It is licensed for the treatment of various recurrent or metastatic cancers, including colorectal cancer, non-squamous non-small-cell lung cancer (NSCLC), glioblastoma, renal cell carcinoma, cervical cancer, epithelial ovarian, fallopian tube, primary peritoneal cancer, and hepatocellular carcinoma [8]. Several randomized controlled trials have demonstrated improved overall survival (OS) and progression-free survival (PFS) with chemotherapy plus bevacizumab compared to chemotherapy alone [9-11]. Despite the benefits described above, high drug costs and health insurance restrictions may be barriers to patients’ efforts to access bevacizumab [12].

Biosimilars, biological drugs similar to the originators developed by different manufacturers, have close similarities to the approved originators in terms of quality, efficacy, and safety. Due to their lower prices, the widespread use of biosimilars is expected to make a significant contribution to reducing healthcare costs and increasing patients’ access to biological drugs [13,14]. Biosimilars have large molecular weights and complex structures, making it difficult to demonstrate their bioequivalence to originators; thus, a clinical trial is required to demonstrate equivalence of efficacy and safety to the corresponding originator, in contrast to small molecule drugs. However, clinical trials often have strict exclusion criteria. Consequently, patients for whom efficacy and safety have not been proven in clinical trials sometimes receive biosimilars in real-world settings.

To date, many types of bevacizumab biosimilars have been approved for use in different countries and regions. ABP 215 (Amgen, Thousand Oaks, CA, USA) was the first bevacizumab biosimilar approved by the U.S. Food and Drug Administration (FDA) (and the second approved in Japan). ABP 215 has been proven through comprehensive analytical characterization to be equivalent to the originator, with minor variations that do not affect the quality, efficacy, or safety of the product [15,16]. In a randomized, double-blind, phase III clinical trial, this biosimilar demonstrated similar efficacy and safety to the originator product in patients with non-squamous NSCLC in combination with carboplatin and paclitaxel [17]. Moreover, according to a systematic review and meta-analysis of 6,416 patients with non-squamous NSCLC in 10 randomized clinical trials, no significant differences in objective response rate, PFS, or OS were observed between biosimilars and originators [18]. Jin et al. [19] demonstrated no significant differences in pharmacokinetics, efficacy, or safety between the bevacizumab biosimilar ABP 215 and the originator as a first-line treatment in patients with metastatic colorectal cancer. In addition, another report has been published comparing clinical outcomes between the bevacizumab biosimilar ABP 215 and an originator product in colorectal cancer patients [20], while there have been no reports to date on the efficacy and safety data for the Japanese population diagnosed with colorectal cancer. Given this background, this study aimed to compare the efficacy, safety, and economics between biosimilar ABP 215 and originator bevacizumab in Japanese patients with colorectal cancer.

## Materials and methods

Study subjects

We conducted a single-center, retrospective, observational cohort study. Electronic medical records from Oita University Hospital, Yufu, Japan, were reviewed to extract data on patients diagnosed with colorectal cancer who received the originator bevacizumab (Avastin) or biosimilar ABP 215 between January 2018 and April 2024. Bevacizumab adoption in our institute shifted from the originator to the biosimilar ABP 215 in October 2021. Patients were excluded if they received only one dose of bevacizumab, switched from the originator to the biosimilar ABP 215 during bevacizumab therapy, or did not complete the two-year observation period and were still receiving bevacizumab therapy as of July 1, 2024. The study was conducted according to the ethical standards of our institute and the tenets of the Declaration of Helsinki, issued in 1975 and revised in 2013. The study was initiated after approval by the Ethics Committee of the Faculty of Medicine, Oita University, Japan (Review Reference Number 2834).

Data collection

The following demographic and background information was collected for each patient: sex, age, height, weight, performance status (PS), line of chemotherapy, history of treatment with molecularly targeted drugs, KRAS gene mutation, the number of bevacizumab doses received, bevacizumab dose intensity (mg/kg), and combined chemotherapy regimen. Body mass index (BMI) was calculated as follows: BMI = weight (kg) / [height (m)]^2^. PS was graded according to the Eastern Cooperative Oncology Group criteria. The following laboratory parameters were monitored during bevacizumab therapy: white blood cell (WBC), hemoglobin, platelets (PLT), neutrophils (Neut), serum albumin, aspartate aminotransferase (AST), alanine aminotransferase (ALT), serum creatinine (Scr), estimated glomerular filtration rate, urine protein, and systolic blood pressure. Follow-up was set for up to two years. Nursing notes at the time of infusion were reviewed to assess infusion-related reactions, defined as symptoms such as redness, wheals, pain, itching, facial pallor, decreased consciousness, discomfort, sweating, chills, and rash during bevacizumab infusion.

Evaluation of efficacy, safety, and economics

Enrolled colorectal cancer patients were divided into two cohorts: those who received only the originator and those who received only the biosimilar ABP 215 until the end of bevacizumab therapy. For efficacy, PFS was assessed over the two-year follow-up period and compared between cohorts. Specifically, PFS was defined as the duration from the initiation of therapy to the occurrence of first recorded disease progression, death from any cause, or censoring (the end of the medical record or end of follow-up period), whichever occurred first. For safety, the following adverse events were evaluated based on the Common Terminology Criteria for Adverse Events (CTCAE) v5.0 [21]: anemia, WBC decreased, Neut decreased, PLT decreased, AST increased, ALT increased, Scr increased, hypoalbuminemia, proteinuria, chronic kidney disease, hypertension, and infusion-related reaction. The incidence of any grade and grade 3-4 adverse events occurring during bevacizumab therapy was compared between cohorts. The rate of patients experiencing infusion-related reactions was also compared. To assess economic benefits, costs were calculated by multiplying each originator and biosimilar’s price by the number of vials used for the biosimilar ABP 215 cohort and then dividing by the number of patients. The selected drug prices were those for Japan in April 2024.

Statistical analysis

SPSS Statistics Version 29 (IBM Corp., Armonk, NY, USA) was used for all statistical analyses. Data are expressed as numbers (%) for categorical and ordinal variables and as medians [interquartile range] for continuous variables. Categorical variables were analyzed using the chi-square goodness-of-fit test. Continuous variables were analyzed using the Mann-Whitney U test. PFS was plotted as a Kaplan-Meier curve, and the log-rank test was used to determine significant differences between these curves. The Cox regression analysis was used to calculate the hazard ratio. Statistical significance was defined as a p-value of <0.05.

## Results

Patient characteristics

**Figure 1:**
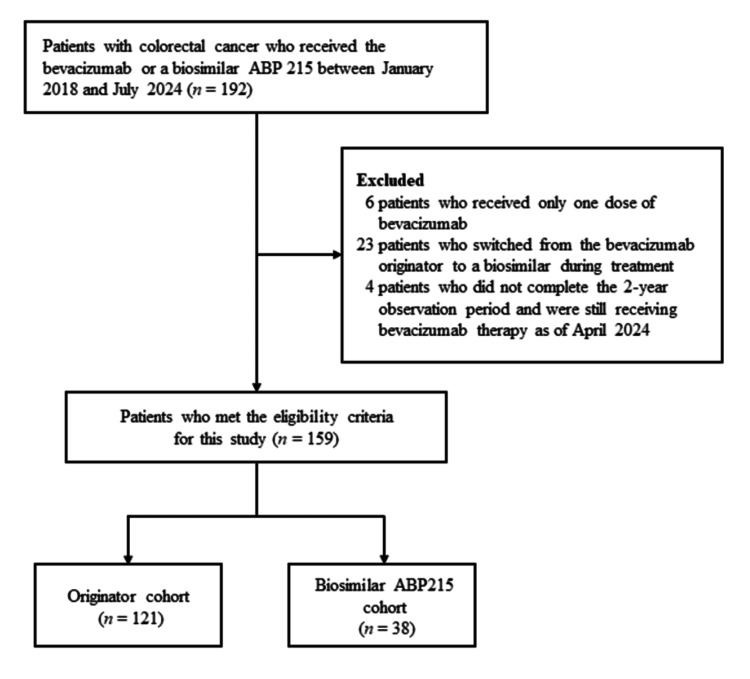
Eligibility flowchart of study participants.

Figure 1 illustrates the eligibility flowchart for the study’s participants. A total of 159 patients were eligible for this study and then divided into the originator cohort (n=121) and the biosimilar ABP 215 cohort (n=38). Table 1 summarizes the clinical characteristics and chemotherapy-related data overall and in both cohorts. The median age was 67.0 years in the originator cohort and 66.0 years in the biosimilar ABP 215 cohort, with slightly more men (57-58%) in both cohorts. Most patients in both cohorts had PS 0 or 1 and received bevacizumab as a first- or second-line chemotherapy. Approximately half of the patients in both cohorts were treated with molecularly targeted agents prior to bevacizumab therapy. The most commonly used chemotherapy regimen in combination with bevacizumab was FOLFIRI (levofolinate, irinotecan, and fluorouracil regimen) in the originator cohort and FOLFIRI and TAS-102 (trifluridine/tipiracil) in the biosimilar ABP 215 cohort. No significant differences were observed between the cohorts for all clinical characteristics and chemotherapy-related data.

**Table 1:** Clinical characteristics and chemotherapy-related data. Data are expressed as numbers (%) for categorical and ordinal variables and as medians [interquartile range] for continuous variables. ^a^Categorical variables were analyzed using the chi-square goodness-of-fit test. ^b^Continuous variables were analyzed using the Mann–Whitney U test. BMI, body mass index; CAPEOX, capecitabine and oxaliplatin regimen; ECOG, Eastern Cooperative Oncology Group; FOLFIRI, levofolinate, irinotecan and fluorouracil regimen; mFOLFOX6, modified levofolinate, fluorouracil, and oxaliplatin regimen; sLV5FU2, fluorouracil and levofolinate regimen; IRIS, irinotecan and S-1 (tegafur/gimeracil/oteracil) regimen; TAS-102, trifluridine/tipiracil

Characteristic	Total (n=159)	Originator (n=121)	Biosimilar ABP 215 (n=38)	p-Value
Age (years)	66.0 [54.5, 72.0]	67.0 [55.0, 71.0]	66.0 [54.3, 73.8]	0.740^b^
Sex, n (%)				
Male	91 (57.2)	69 (57.0)	22 (57.9)	>0.999^a^
Female	68 (42.8)	52 (43.0)	16 (42.1)	
BMI at baseline (kg/m^2^)	22.5 [20.8, 25.0]	22.9 [20.6, 25.2]	22.4 [21.0, 24.2]	0.514^b^
ECOG performance status, n (%)				
0	102 (64.2)	77 (63.6)	25 (65.8)	0.923^a^
1	48 (30.2)	36 (29.8)	12 (31.6)	
2	8 (5.0)	7 (5.8)	1 (2.6)	
3	1 (0.6)	1 (0.8)	0 (0.0)	
Lines of chemotherapy, n (%)				
1	83 (52.2)	64 (52.9)	19 (50.0)	0.712^a^
2	54 (34.0)	41 (33.9)	13 (34.2)	
3	17 (10.7)	13 (10.7)	4 (10.5)	
4	4 (2.5)	2 (1.7)	2 (5.3)	
5	1 (0.6)	1 (0.8)	0 (0.0)	
History of treatment with molecular targeted agents, n (%)				
-	87 (54.7)	68 (56.2)	19 (50.0)	0.677^a^
+	72 (45.3)	53 (43.8)	19 (50.0)	
*KRAS* mutation status, n (%)				
Unknown	113 (71.1)	90 (74.4)	23 (60.5)	0.106^a^
Known positive	46 (28.9)	31 (25.6)	15 (39.5)	
Number of doses	12.0 [7.0, 17.0]	13.0 [8.0, 17.0]	10.0 [5.0, 14.8]	0.064^b^
Dose intensity (mg/kg)	5.00 [4.94, 7.38]	5.00 [4.94, 5.08]	5.00 [4.96, 7.43]	0.385^b^
Combined chemotherapy regimen				
Capecitabine	18 (11.3)	10 (8.3)	8 (21.1)	
CAPEOX	26 (16.4)	20 (16.5)	6 (15.8)	
FOLFIRI	46 (28.9)	37 (30.6)	9 (23.7)	
mFOLFOX6	37 (23.3)	34 (28.1)	3 (7.9)	
sLV5FU2	3 (1.9)	3 (2.5)	0 (0.0)	
IRIS	10 (6.3)	7 (5.8)	3 (7.9)	
TAS-102	19 (11.9)	10 (8.3)	9 (23.7)	

Efficacy

**Figure 2:**
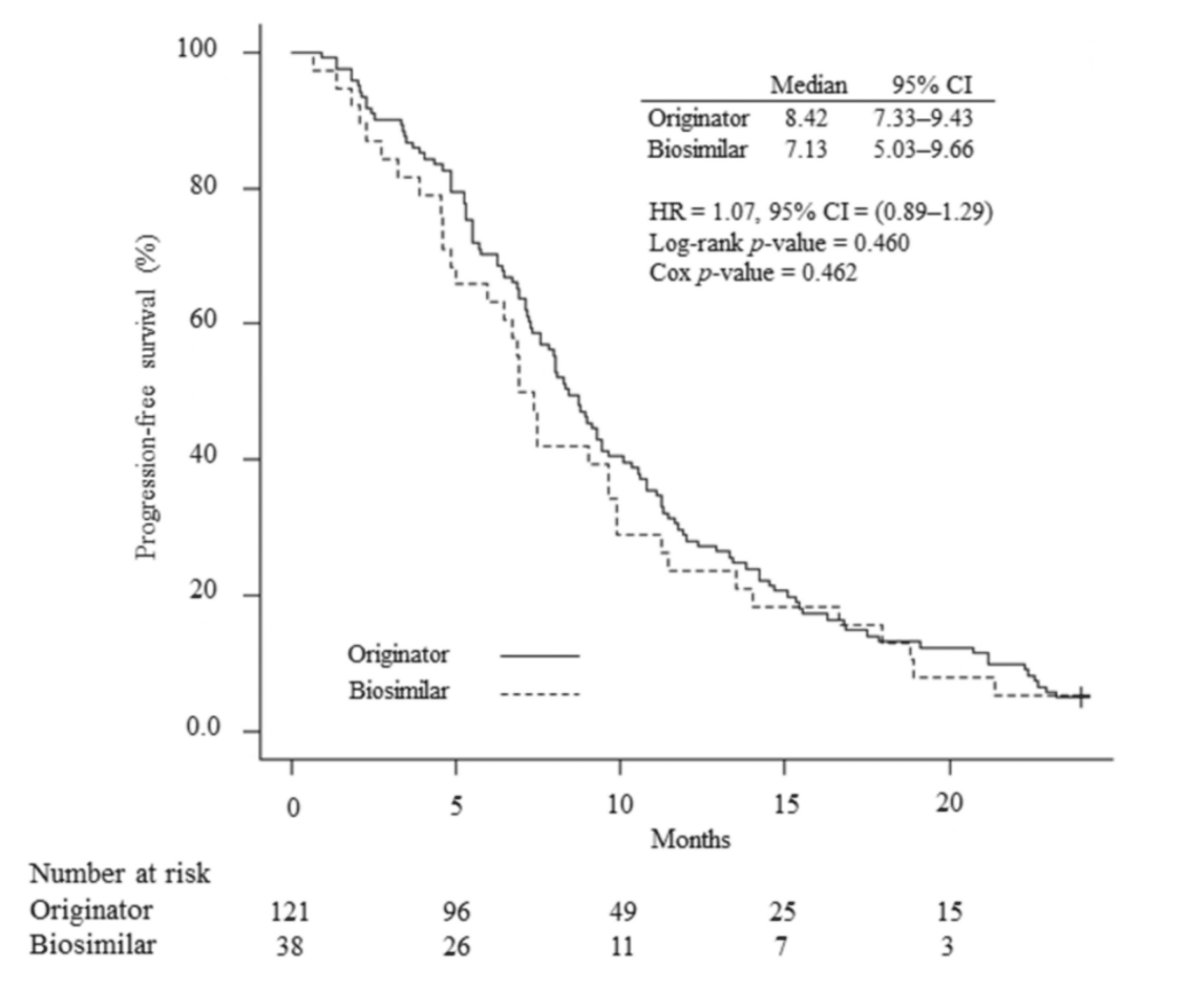
Kaplan-Meier curves for progression-free survival. The number of patients at risk for each treatment arm is shown below the survival curves. The log-rank test was used to determine significant differences between these curves. The hazard ratio was calculated using Cox regression analysis.

Figure 2 shows the Kaplan-Meier curves for PFS during two years of follow-up in the originator and biosimilar ABP 215 cohorts. Two-year PFS was approximately 10% in both cohorts. Median PFS was 8.41 months (95% CI, 7.33-9.43) in the originator cohort and 7.13 months (95% CI, 5.03-9.66) in the biosimilar ABP 215 cohort. Based on the Cox regression model, the estimated hazard ratio for biosimilar and originator comparison was 1.07 (95% CI, 0.89-1.29). The log-rank test revealed no significant differences in PFS between the cohorts (p=0.460).

Safety

Table 2 summarizes the adverse events of any grade or grade 3-4 that occurred during bevacizumab therapy in both cohorts. The most common adverse events in both cohorts were hypoalbuminemia, followed by anemia and a decrease in neutrophil count. The most common grade ≥3 serious adverse events in both cohorts were related to myelosuppression, such as anemia and neutropenia. Approximately 70% and 40% of patients in both cohorts developed proteinuria and hypertension, respectively, which are typical adverse events related to bevacizumab. Only one patient in the originator cohort developed grade ≥3 proteinuria. The incidence rates of any and grade ≥3 adverse events did not differ significantly between cohorts.

**Table 2:** Any grade or grade 3-4 adverse events that occurred during bevacizumab therapy. Data are expressed as numbers (%). All adverse events were graded using CTCAE v5.0. ^a^Continuous variables were analyzed using the Mann–Whitney U test.

Adverse events	Originator (n=121)		Biosimilar ABP 215 (n=38)		p-Value
	Any grade	Grade 3-4	Any grade	Grade 3-4	
Total of any grade	121 (100)		38 (100)		>0.999^a^
Total of grade 3-4		47 (42.7)		12 (38.7)	0.837^a^
Blood and lymphatic system disorders, n (%)					
Anemia	101 (83.5)	9 (7.4)	28 (73.7)	5 (13.2)	
Investigations, n (%)					
White blood cell count decreased	74 (61.2)	21 (17.4)	17 (44.7)	6 (15.8)	
Neutrophil count decreased	100 (82.6)	50 (41.3)	27 (71.1)	14 (36.8)	
Platelet count decreased	95 (78.5)	6 (5.0)	23 (60.5)	1 (2.6)	
Alanine aminotransferase increased	65 (53.7)	6 (5.0)	20 (52.6)	0 (0.0)	
Aspartate aminotransferase increased	92 (76.0)	6 (5.0)	26 (68.4)	2 (5.3)	
Creatinine increased	41 (33.9)	0 (0.0)	11 (28.9)	0 (0.0)	
Metabolism and nutrition disorders, n (%)					
Hypoalbuminemia	108 (89.3)	1 (0.8)	37 (97.4)	2 (5.3)	
Renal and urinary disorders, n (%)					
Proteinuria	55 (45.5)	1 (0.8)	19 (50.0)	0 (0.0)	
Chronic kidney disease	81 (66.9)	3 (2.5)	28 (73.7)	0 (0.0)	
Vascular disorders, n (%)					
Hypertension	48 (39.7)	0 (0.0)	14 (36.8)	0 (0.0)	
Injury, poisoning, and procedural complications, n (%)					
Infusion-related reaction	0 (0.0)	0 (0.0)	0 (0.0)	0 (0.0)	

Economic outcomes

**Figure 3:**
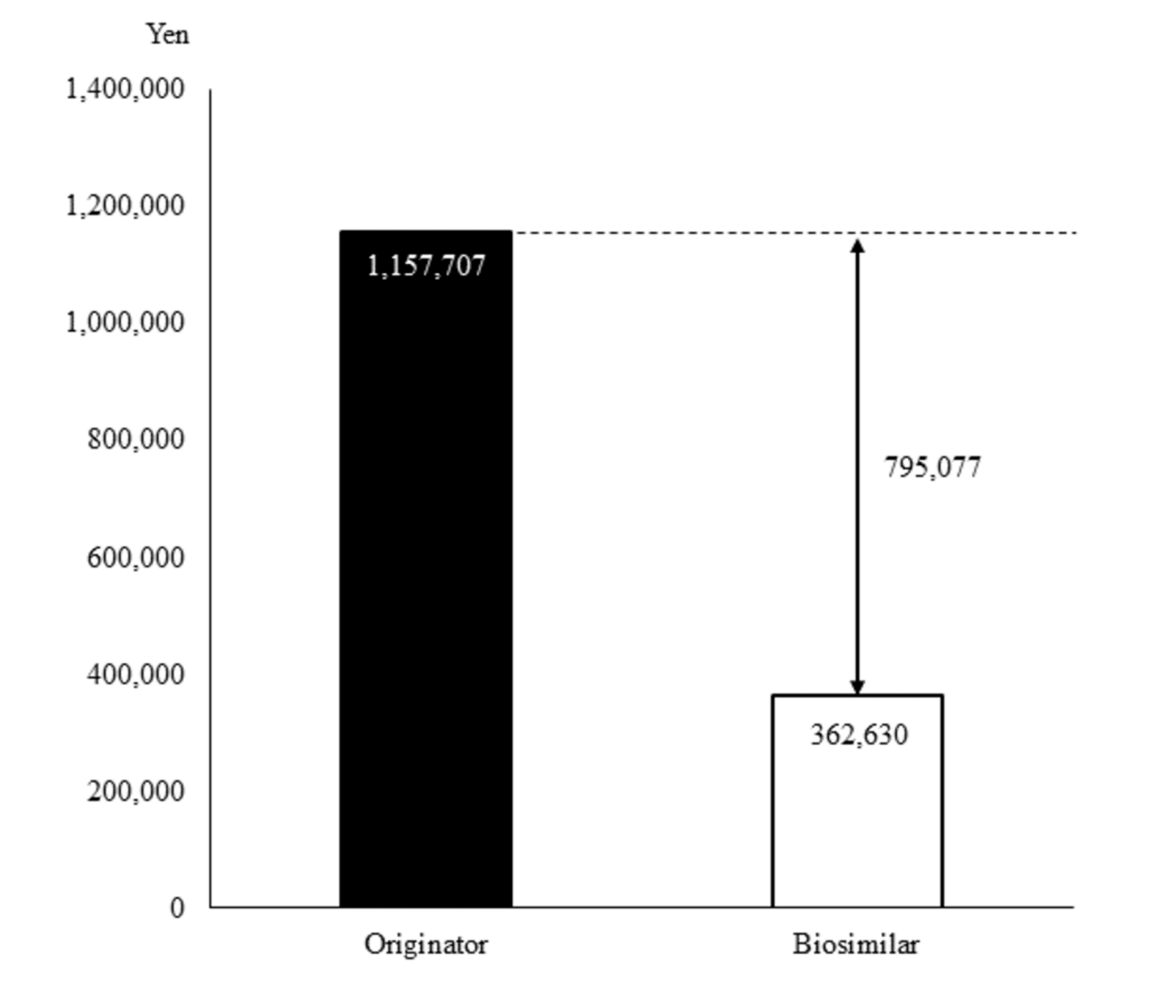
Economic benefits of switching to biosimilars. Costs were calculated by multiplying each originator and biosimilar’s price by the number of vials used for the biosimilar cohort and then dividing by the number of patients. The selected drug prices are those for Japan in April 2024.

Figure 3 compares the mean cost per patient for bevacizumab therapy between the originator and biosimilar ABP 215 cohorts. The median number of doses and dose intensity were 13.0 doses and 5.0 mg/kg in the originator cohort and 10.0 doses and 5.0 mg/kg in the biosimilar ABP 215 cohort (Table 1). The mean costs per patient were 1,157,707 and 362,630 Japanese yen in the originator and biosimilar ABP 215 cohorts, respectively, indicating a potential savings of approximately 800,000 per patient with biosimilar ABP 215 use.

## Discussion

Bevacizumab is one of the main anticancer treatment options for prolonging life and relieving symptoms in patients with unresectable advanced or recurrent colorectal cancer. Despite its efficacy advantages, the high cost of the drug has been a barrier to its use. In recent years, bevacizumab biosimilars have been launched and reported as non-inferior to the originator product in terms of their therapeutic effects in patients with NSCLC and colorectal cancer [15-20,22]. To date, there have been no reports comparing the efficacy and safety of originator and biosimilar ABP 215 bevacizumab in Japanese colorectal cancer patients. Our retrospective study demonstrated no significant differences in efficacy and safety between the two formulations in Japanese colorectal cancer patients but did demonstrate a savings of approximately 800,000 Japanese yen per patient.

The median PFS was 8.41 months in the originator cohort and 7.13 months in the biosimilar ABP 215 cohort (hazard ratio = 1.07 [95%CI, 0.89-1.29], p=0.462), indicating no significant difference in efficacy between the cohorts. Several previous clinical trials, retrospective studies, and a meta-analysis reported similar efficacy between originator and biosimilar bevacizumab in colorectal cancer patients [19-20,22-24], corroborating our findings. However, the median PFSs in the originator and biosimilar cohorts were slightly shorter than those noted in Romera et al. (11.1 and 10.8 months) [22] and Jin et al. (8.6 and 14.1 months) [19]. This may be, in part, attributed to the difference in the number of chemotherapy lines compared to previous reports, which focused on the first-line setting. Indeed, median PFS in both the originator and biosimilar cohorts was longer in patients receiving first-line bevacizumab (11.29 [95%CI, 8.74-12.91] and 7.46 [95%CI, 5.03-11.43] months, p=0.484) than in those receiving second-line or later bevacizumab (6.70 [95%CI, 5.29-8.05] and 6.74 [95%CI, 3.91-9.89] months, p=0.902) (data not shown). In contrast, a phase III randomized clinical trial that did not specify the line for bevacizumab in patients with metastatic colorectal cancer showed a median PFS of 7.0 months in the originator cohort and 7.7 months in the biosimilar cohort, which supports our findings [23].

This study evaluated any-grade adverse events during bevacizumab therapy using CTCAE v5.0 and identified hypoalbuminemia as the most common adverse event in both cohorts, occurring in approximately 90% of patients. This may be because this study included advanced or recurrent colorectal cancer, with many patients developing cachexia. Cachexia is a metabolic syndrome secondary to underlying diseases, such as cancer and chronic kidney disease, and is observed in approximately 50-80% of patients with advanced cancer [25]. During cancer cachexia, nutritional status becomes poor, and the synthesis of acute phase proteins increases, resulting in hypoalbuminemia due to reduced albumin synthesis in many patients [26]. Therefore, the hypoalbuminemia in both groups would be due to the primary disease and not an adverse event caused by bevacizumab.

In contrast to the above, most grade 3 or higher adverse events in both cohorts were related to bone marrow suppression, such as neutropenia and anemia, which is in line with the findings of a previous study [22]. Bevacizumab is used in combination with fluoropyrimidine-based chemotherapy to treat colorectal cancer. Indeed, patients enrolled received bevacizumab primarily with FOLFIRI, mFOLFOX6 (modified levofolinate, fluorouracil, and oxaliplatin regimen), or CAPEOX (capecitabine and oxaliplatin regimen) for the originator cohort and FOLFIRI, TAS-102, or capecitabine for the biosimilar ABP 215 cohort. The cytotoxic anticancer drugs in these regimens damage not only cancer cells but also actively dividing cells, such as bone marrow cells, often resulting in bone marrow suppression. Bevacizumab would, therefore, not be responsible for these types of adverse events. The type of combination chemotherapy regimen did not differ significantly between the cohorts, which may have led to no difference in the incidence of anemia and neutrophil decline.

Gastrointestinal perforation, surgical and wound healing complications, thromboembolism, and hemorrhage have been reported as the most serious adverse effects of bevacizumab, but none was observed in either cohort according to the electronic medical records examined in this study [8]. Proteinuria and hypertension are other common adverse events. Bevacizumab blocks the binding of VEGF to its receptor, thereby reducing VEGF receptor signaling. This contributes to systemic vasoconstriction and increased peripheral resistance due to endothelial dysfunction and, possibly, other vascular abnormalities, leading to hypertension [27]. In addition, inhibited VEGF signaling may alter renal function and structure, resulting in inadequate renal sodium excretion and fluid overload, which may lead to proteinuria [27]. In this study, 39.7% and 36.8% of patients treated with originator and biosimilar ABP 215 bevacizumab, respectively, developed hypertension. According to a systematic review and meta-analysis, 36% of patients treated with originator bevacizumab developed hypertension, which would support our study results.

Our single-center, retrospective, observational study has several limitations. First, as a single-center study, the number of patients was small, with only 38 patients enrolled in the biosimilar ABP 215 cohort. As a result, a small but statistically significant difference may have been missed. Second, it cannot be concluded that there was no difference in OS between the cohorts, as efficacy was only assessed by PFS. Third, as a retrospective study, patients’ backgrounds and clinical characteristics were not completely consistent between cohorts. However, no significant differences were observed between the cohorts in factors that could potentially affect efficacy and/or safety. Therefore, propensity score matching for background factors was not performed.

## Conclusions

This was the first study to verify that a bevacizumab biosimilar ABP 215 is as effective and safe as the originator product in real-world Japanese colorectal cancer patients. Our retrospective study demonstrated no significant differences in efficacy and safety between the two formulations but did demonstrate a saving in medical costs in the biosimilar ABP 215 cohort. With the findings of no differences in efficacy and safety, the active use of the bevacizumab biosimilar ABP 215 product is also recommended in Japan from the perspective of reducing medical costs.
